# Utility of angiography–physiology coregistration maps during percutaneous coronary intervention in clinical practice

**DOI:** 10.1007/s12928-020-00668-0

**Published:** 2020-06-07

**Authors:** Akiko Matsuo, Takeru Kasahara, Makoto Ariyoshi, Daisuke Irie, Koji Isodono, Yoshinori Tsubakimoto, Tomohiko Sakatani, Keiji Inoue, Hiroshi Fujita

**Affiliations:** Department of Cardiology, Japanese Red Cross Kyoto Daini Hospital, 355-5 Haruobi-cho, Kamanzadorimarutamachi, Kamigyo-ku, Kyoto, 602-8026 Japan

**Keywords:** Instantaneous wave-free ratio, Physiological map, Pullback tracing, Coregistration

## Abstract

This study aimed to evaluate the utility and feasibility of physiological maps coregistered with angiograms using the pullback of a pressure guidewire with continuous instantaneous wave-free ratio (iFR) measurements. iFR pullback was obtained for 70 lesions from 70 patients with stable angina pectoris using SyncVision (Philips Corp.). Physiological maps were created, whereby the post-intervention iFR (post-iFR) was predicted as iFRpred. The iFR gap was defined if the difference between the iFRpred and post-iFR was ≥ 0.3. The lesion morphology changed from that during the physiological assessment to that during the angiographic assessment in 26 lesions (37.1%). In particular, 22.6% of angiographic tandem lesions changed to physiological focal lesions. The mean pre-intervention iFR, post-iFR, and iFRpred were 0.73 ± 0.17, 0.90 ± 0.06, and 0.93 ± 0.05, respectively. The mean difference between the iFRpred and post-iFR was 0.029 ± 0.099, with 95% limits of agreement of -0.070–0.128. iFR gaps occurred in 28 patients (40%). Notably, a new iFR gradient causing a ≥ 0.03 iFR drop after stenting occurred in 11 (15.7%) cases. The study patients were divided into two groups according to biases between post-iFR and iFRpred < 0.03 (good concordance group) or ≥ 0.03 (poor concordance group). The pre-intervention heart rate was the only independent predictor of poor concordance (odds ratio, 0.936; 95% confidence interval 0.883–0.992; *p* = 0.027). Physiological maps under resting conditions may contribute to a reduction in unnecessary stent placements without missing lesions requiring treatment. However, the predictive accuracy of post-iFR performance in the present study was slightly lower than that in the previous reports.

## Background

Fractional flow reserve (FFR) has been established as a diagnostic method for ischemia-inducing epicardial disease in patients with stable angina pectoris and is part of the current guidelines [1–4]. However, when treating subset lesions with percutaneous coronary intervention (PCI), especially tandem or diffuse lesions, FFR-guided PCI is challenging, because with hyperemic flow, the fluid dynamic interaction between stenoses alters their relative severity, which complicates the interpretation of each FFR value. This creates uncertainty regarding the lesion that should be treated [5–7]. However, resting flow is maintained at a constant and stable level until critical stenosis develops, even across a tandem lesion [8, 9]. The instantaneous free-wave ratio (iFR) has recently emerged as a new diagnostic tool [10], and rapidly accumulated robust evidence shows equivalence to FFR in terms of the benefits of revascularization [7, 11–13]. Based on the aforementioned peculiarity of resting flow [8, 9], the resting flow index, including iFR, is considered suitable for assessing tandem lesions. Current cutting-edge technology makes it possible to coregister the iFR pullback curve with angiography, and a diagnostic software, SyncVision (Philips/Volcano, Amsterdam, the Netherlands), has been commercially introduced for the precise assessment of stenosis severity, providing comprehensible information of both angiography and physiology simultaneously to guide PCI. We evaluated ischemia-induced coronary artery disease in clinical practice including angiographic tandem lesions before and after PCI using SyncVision.

## Methods

### Study population

We investigated 70 coronary lesions from 70 patients who had angiographic stenoses with a stenosis diameter of 40–80% on visual estimation and who were amenable to PCI, including angiographic tandem or diffuse lesions with FFR values of 0.8 or less or iFRs of 0.89 or less. Patients were excluded if the drift in the iFR was greater than 0.03, regardless of repeated measurements. Patients with coronary artery bypass graft vessels, culprit vessels of acute coronary syndrome, congestive heart failure, tortuous vessels, and hard calcified lesions requiring a rotablator to deliver stents were excluded. Physiological measurements were acquired and PCI was performed between July 2018 and July 2019 at the Japanese Red Cross Kyoto Daini Hospital. The study was approved by the institutional review board and all patients provided written informed consent.

### Angiographic parameters and angiographic lesion classification

For quantitative coronary angiography (QCA), an automated edge-detection algorithm was used; additionally, the offline analysis was performed by examiners who were unaware of the results of the pressure wire examination. Conventional angiograms were assessed using an offline QCA system (QAngio XA 7.3, Medis Medical Imaging Systems, The Netherlands). The reference diameter (RD), minimum lumen diameter (MLD), and lesion length (LL) were measured using an edge-detection system, and the % diameter stenosis (%DS) was subsequently calculated. Notably, angiographic lesions are classified into focal, tandem, and diffuse lesions. Focal lesions were defined as lesions with > 50% stenosis and lengths < 20 mm, tandem lesions based on angiography as 2 separate lesions with > 50% stenosis in the same coronary artery separated by an angiographically normal segment, and diffuse lesions as lesions with significant stenoses of ≥ 20 mm, respectively. Angiographic PCI success was defined as < 30%DS in the presence of thrombolysis in myocardial infarction grade 3.

### Procedure and analysis of physiological maps of virtual PCI

Intracoronary nitrates (200 mg) were administered to all patients before coronary angiography. Pressure wire normalization was performed at the coronary ostia before recording using 0.014-inch pressure-tipped wires (Prestige guidewire PLUS/Verrata guidewire PLUS; Philips/Volcano, Amsterdam, The Netherlands). A pressure wire was advanced to the distal segment of the target artery, ensuring that the sensor position was at least 20 mm distal to the most distant lesion in question and fluoroscopically stored. FFR values were then measured during pharmacological hyperemia. Hyperemia was induced by continuous intravenous administration of adenosine at 150 µg/kg/min or intracoronary administration of nicorandil (2 mg). After hyperemia subsided, the iFR was remeasured to confirm that its value had remained unchanged. Resting pressure wire pullback was then manually performed while maintaining a constant speed until the pressure sensor reached the ostium of the coronary artery. The presence of pressure wire drift was identified at the end of all the iFR measurements.

We previously investigated 74 patients with coronary diseases to assess the recovery time of pharmacological hyperemia following intracoronary administration of nicorandil or continuous intravenous administration of adenosine. In this study, we first measured the iFRs and subsequently measured FFR values based on pharmacological hyperemia. Finally, the iFRs were remeasured when their values returned to the previous values. The recovery time was defined as the time between the measurement of FFR values and remeasurement of the iFR. As a result, the median and average recovery times were 203.5 s and 207.4 ± 71.4 s, respectively. Based on the results, we measured post-intervention iFRs (post-IFRs) for longer than 5 min after the final procedure. After the pullback, angiography was performed on the same projection, in which automatic coregistration of the iFR pullback curve with the angiogram was performed using customized software (SyncVision, Philips/Volcano, Amsterdam, The Netherlands). iFR maps were created using SyncVision, whereby the strategy of stent implantation was implemented at the discretion of the treating physician according to the following virtual PCI. PCI was performed using conventional guidewires dedicated to PCI.

On the physiological maps, a physiologically significant lesion was defined as a lesion with an iFR gradient ≥ 0.03 (3 iFR units or more) based on previously reported error ranges [14, 15].

**Fig. 1:**
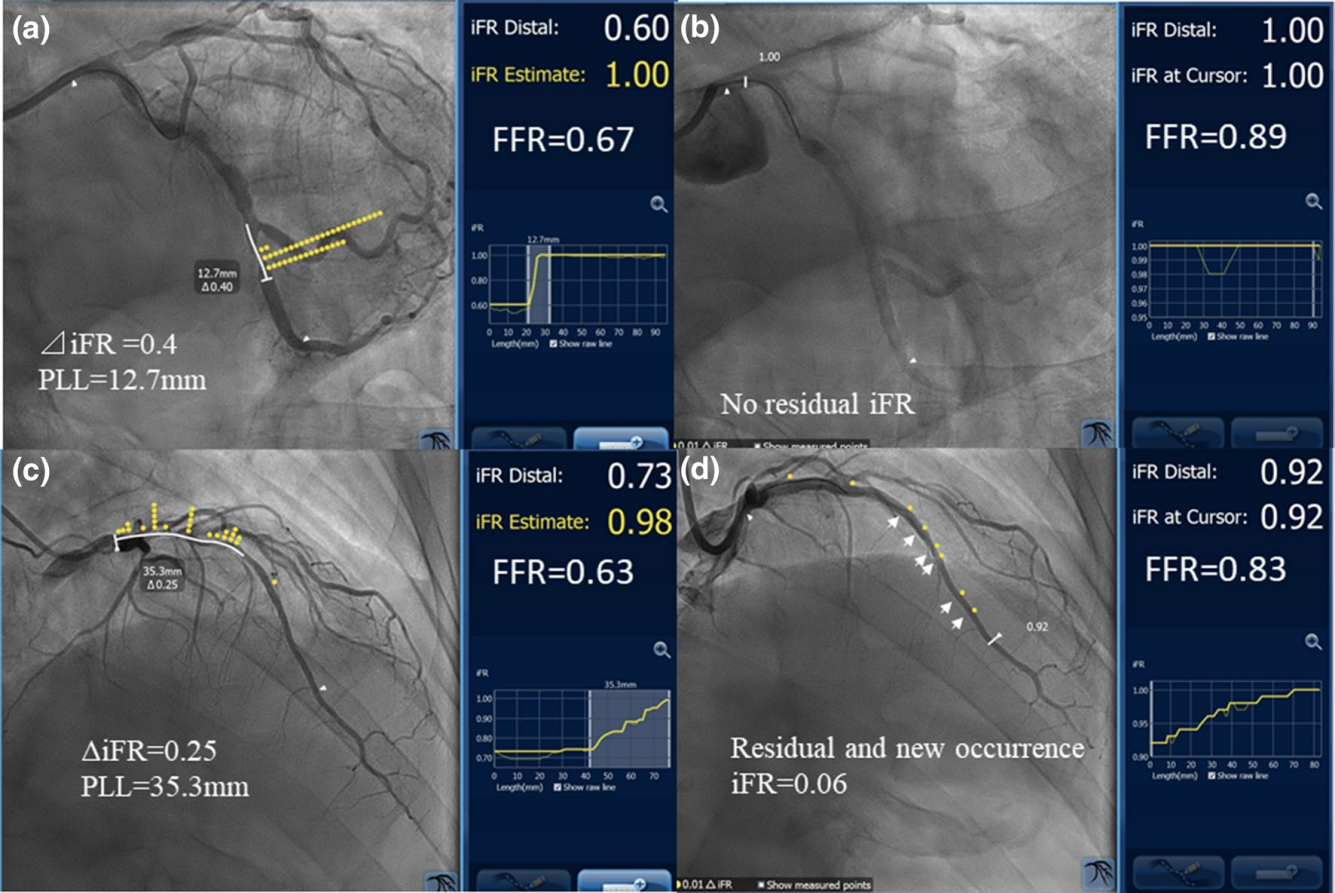
Representative physiological maps of cases before and after PCI generated using SyncVision. Case 1: the left coronary angiogram revealed a tandem lesion in the LCx. However, the iFR physiological map showed a physiologically significant lesion in the distal portion with an iFR of 0.60 and an FFR value of 0.67. Virtual PCI predicted the post-intervention iFR, which was defined as the iFRpred, as 1.0, assuming that stent implantation could remove an iFR drop of 0.4 (**a**). After stenting in the distal segment, the iFR increased to 1.0, as predicted using SyncVision, while the FFR value was 0.89 (**b**). This case was assigned to the good concordance group. Case 2: the left coronary angiogram revealed a focal stenotic lesion extending from the distal portion of the LMT to the proximal portion of the LAD, and the iFR map also showed a focal physiologically significant segment corresponding to angiographical stenosis with an iFR of 0.73 and an FFR value of 0.63. Virtual PCI was performed with crossover LMT stenting, which eliminated an iFR drop of 0.25, and the iFRpred was 0.98. After LMT–LAD crossover stenting according to the iFR map, the post-intervention iFR was 0.92, which was lower than expected, and the bias was 0.03 or greater. As a result, the iFR gap was 0.06, and a new iFR drop occurred in the distal reference vessel with a significant gradient in the iFR, which had been a physiological normal segment before stenting (**d**). This case was assigned to the poor concordance group. *PCI* percutaneous coronary intervention, *LCx* left circumflex coronary artery, *iFR* instantaneous wave-free ratio, *FFR* fractional flow reserve, *iFRpred* predicted IFR, *LMT* left main trunk, *LAD* left anterior descending artery

Physiological lesion classification based on the pullback curves was as follows: focal, tandem, gradual, and a combination of gradual and focal patterns. A focal pattern was defined as a physiologically significant lesion with a length of < 20 mm. A tandem lesion pattern was defined as more than two separate lesions with physiologically significant lesions in the same coronary artery, separated by physiologically insignificant segments, which were defined as segments with iFR gradients < 0.03. Moreover, in the tandem lesion pattern, the length separating physiologically significant lesions was defined as the physiological interstenosis length. A gradual pattern was defined as the length of a physiologically significant lesion that was 20 mm or longer and close to the line passing through the start and end points without an inflection point [16] (Fig. 1d). The lesions were manually selected and defined based on physiologically significant LLs and the physician intended to remove the iFR gradient by stent implantation. Meanwhile, a drop in the iFR in the selected lesions was automatically displayed, which was defined as delta iFR. SyncVison automatically calculated the post-iFR that would be expected if the stenosis was completely removed by intervention. The predicted iFR (iFRpred) was defined as the pre-intervention iFR plus delta iFR (Fig. 1a, c). In the actual PCI, optimal stent implantation was performed according to the minimum expansion index [17] or multicenter ultrasound stenting in coronaries criteria [18] as much as possible. After PCI, automatic coregistration of the iFR pullback curve with the angiogram was repeatedly performed. Based on the previous studies, which demonstrated mean biases of 0.016 [14] and 0.010 [15], it could predict the post-iFR within 2 ± 1% error [14] and 1.4 ± 0.5% error [15]. The iFR gap was defined when the post-iFR was lower than iFRpred by greater than 0.03 and the location of the iFR gap was identified based on the post-iFR pullback (Fig. 1b, d). A new iFR occurrence was defined when a significant gradient in the iFR (3 iFR units or more) occurred in the segment that had been a physiologically normal segment before stenting (Fig. 1d, arrows).

### Intravascular images and image analysis

In the present study, intravascular ultrasound (IVUS), frequency-domain optical coherence tomography (OCT), or optical frequency-domain imaging (OFDI) was performed using a commercially available system (Boston Scientific, Natick, MA, USA, FastView, Terumo, Tokyo, Japan, or C7 Dragonfly, Abbott Vascular, Santa Clara, CA, USA). The choice of imaging device was made at the operator’s discretion. The quantitative evaluation of intravascular images included the minimal lumen area (MLA), proximal and distal reference lumen area (ref LA), LL, and minimal stent area (MSA).

### Study and statistical analysis

Continuous variables are presented as mean ± standard deviation and categorical variables are presented as numbers and percentages. Continuous agreement between iFRpred and post-iFR was analyzed using the Bland–Altman method. Linear regression analyses were used to investigate the differences between iFRpred and post-iFR. The study population was divided into two groups: a good concordance group consisting of patients with acceptable post-iFRs, wherein the difference between the iFRpred and post-iFR was < 0.03, and a poor concordance group consisting of patients with a difference of 0.3 or greater. Regarding the comparison between the two groups, the association between continuous variables was assessed using the unpaired *t* test and categorical variables were compared using the Chi-square test. For repeated measurements within a group, the paired *t *test was used. A two-way analysis of variance (ANOVA) was used to understand whether there were any differences in the hemodynamic parameters after PCI between the two groups. Pearson’s correlation coefficient was used to evaluate the relationship between two continuous variables. In terms of multivariate analysis to assess predictors of disagreement between the iFRpred and post-iFR, logistic regression was used for categorical variables (poor concordance group). Meanwhile, the aforementioned cut-off value for disagreement between the iFRpred and post-iFR was not authentic and the dichotomy had statistically inherent risks. Therefore, we considered the quantification of the discrepancy, the difference between the iFRpred and post-iFR, as a dependent variable. Structural equation modeling (SEM) was used for multivariate analysis to understand the complex relationships between multiple independent variables and one dependent variable. All statistical analyses were performed using SPSS 26.0 and Amos (SPSS Inc., Chicago, IL, USA).

## Results

### Study demographics

**Fig. 2:**
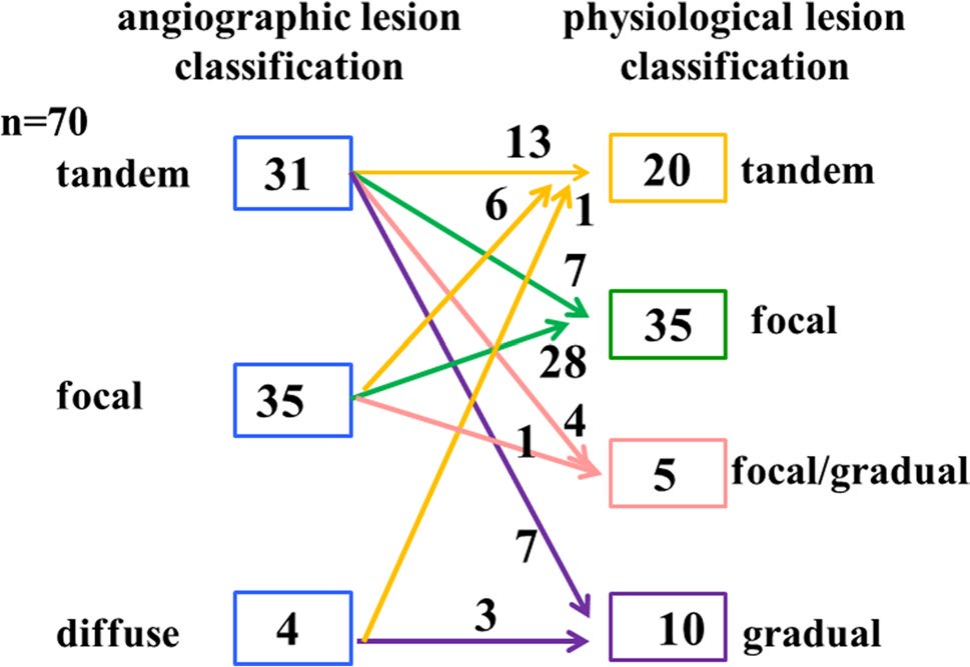
Changes in angiographic lesion classification using SyncVision. Angiographic lesion classification changed to other types of physiological lesion classifications in 26 (37%) of the total lesions

The mean age of the patients was 72 ± 10.4 years, and 75.7% were men. Hypertension was present in 62 (88.6%), diabetes mellitus in 29 (41.4%), dyslipidemia in 53 (75.7%), and chronic kidney disease in 30 (42.9%) patients, respectively; 37 (52.9%) patients were smokers. Baseline angiographic lesion classifications were tandem lesions in 31 (44.3%), focal lesions in 35 (50%), and diffuse lesions in 4 patients (5.7%), respectively. After analysis of the pullback curve of the iFR by SyncVision, 26 lesions (37.1%) changed to other types; physiological lesion classification demonstrated a tandem lesion pattern in 20 (28.6%), focal pattern in 35 (50%), combined focal and gradual pattern in 5 (7.1%), and a gradual pattern in 10 (14.3%) patients, respectively. The most common reclassified subset of lesions were angiographic tandem lesions; that is, 18 (58.1%) tandem lesions changed to other types: 7 lesions changed to a focal pattern, 4 to a combined focal and gradual pattern, and 7 to a gradual pattern. Among the angiographic focal lesions (20%), 6 changed to a tandem lesion pattern and 1 to a combined focal and gradual pattern. One of the four angiographic diffuse lesions changed to a tandem lesion pattern (Fig. 2). All patients achieved angiographic PCI success without experiencing the stent accordion phenomenon. Despite angiographic success, 11 patients (15.7%) could not achieve optimal stenting, wherein coronary stents with a diameter of 2.5 mm were implanted for small vessels in all 11 patients. New ischemic electrocardiography changes or the development of new pathological Q waves were not observed immediately after PCI or on the following day.

### Relationship between actual iFR and predicted iFR after PCI

**Fig. 3:**
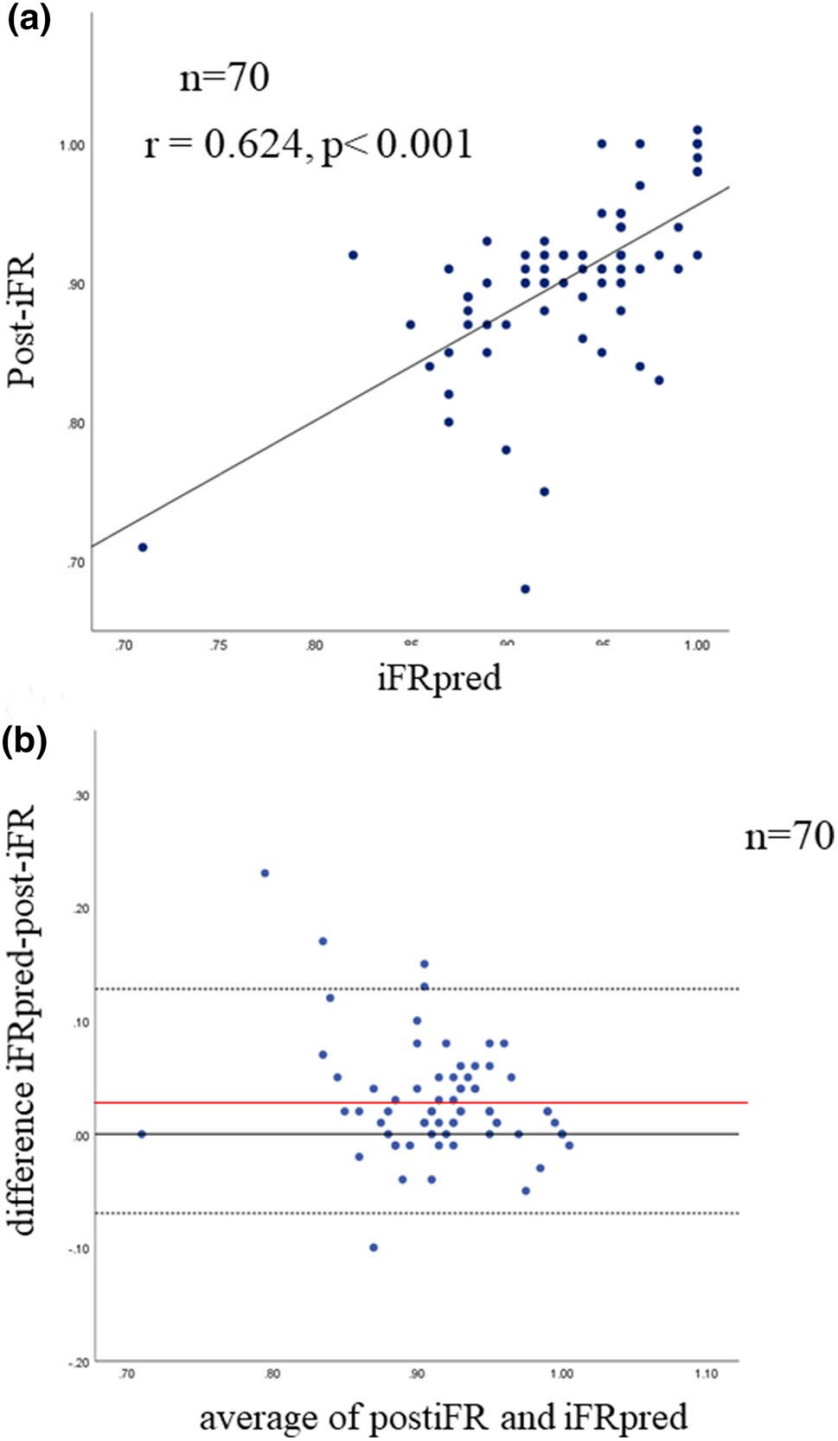
Scatter plot showing the relationship between iFRpred and actual post-iFR (**a**). Bland–Altman plots of differences against the means are displayed. The zero line is displayed in black. The mean bias is represented by the solid red line (with the 95% confidence interval represented by the dashed black line) (**b**). *post-iFR* post-intervention instantaneous wave-free ratio, *iFRpred* predicted instantaneous wave-free ratio

The mean pre-iFR and FFR values were 0.73 ± 0.17 and 0.67 ± 0.09, respectively. The heart rate (HR) was 70.2 ± 11.1 beats/min, and the systolic and diastolic blood pressures were 136.7 ± 23.3 and 67.3 ± 13.8 mmHg, respectively, before PCI. SyncVision was used for calculations as described previously. Also, the mean iFRpred was found to be 0.93 ± 0.05. After PCI, the mean post-iFR and FFR values increased to 0.90 ± 0.06 and 0.84 ± 0.07, respectively. The HR and systolic and diastolic BP were 70.9 ± 0.16 beats/min, 136.6 ± 23.2, and 69.6 ± 15.0 mmHg, respectively. Post-iFR had a significantly positive correlation with iFRpred (*r* = 0.624, *p* < 0.001) (Fig. 3a). The Bland–Altman plot demonstrated that the average difference between the post-iFR and iFRpred was 0.029 ± 0.099, with 95% limits of agreement of − 0.070 to 0.128 (Fig. 3b).

**Fig. 4:**
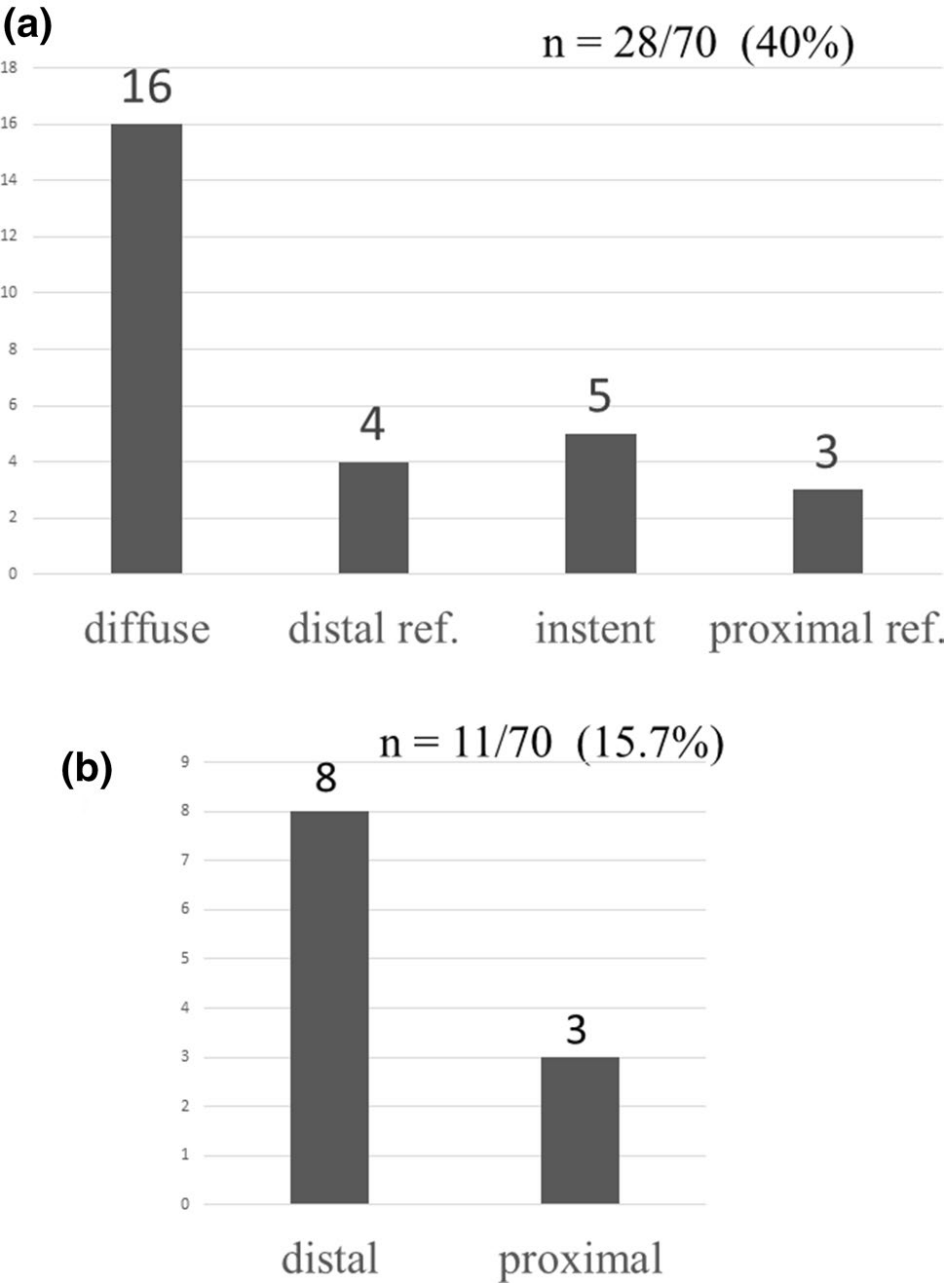
Distribution of iFR gap locations after stent implantation. The iFR gap was most commonly located along the vessel length (**a**). Distribution of new iFR occurrences after coronary intervention (**b**). A new iFR gradient after stenting occurred in 11 patients (15.7%) and was commonly distal to the stent. *distal ref.* distal reference vessel, *proximal ref.* proximal reference vessel, *iFR* instantaneous wave-free ratio

An iFR gap occurred in 28 (40%) study patients and the most common location was in diffuse lesions, in which the iFR gap was spread along the artery length, showing a gradual pattern of pullback tracing (Fig. 1d). The distribution of the locations of the iFR gaps is shown in Fig. 4a. New iFR occurrences were observed in 11 (15.7%) patients, of which 8 occurred distal to the stent (Fig. 4b).

### Discrepancy between post-iFR and iFRpred

**Fig. 5:**
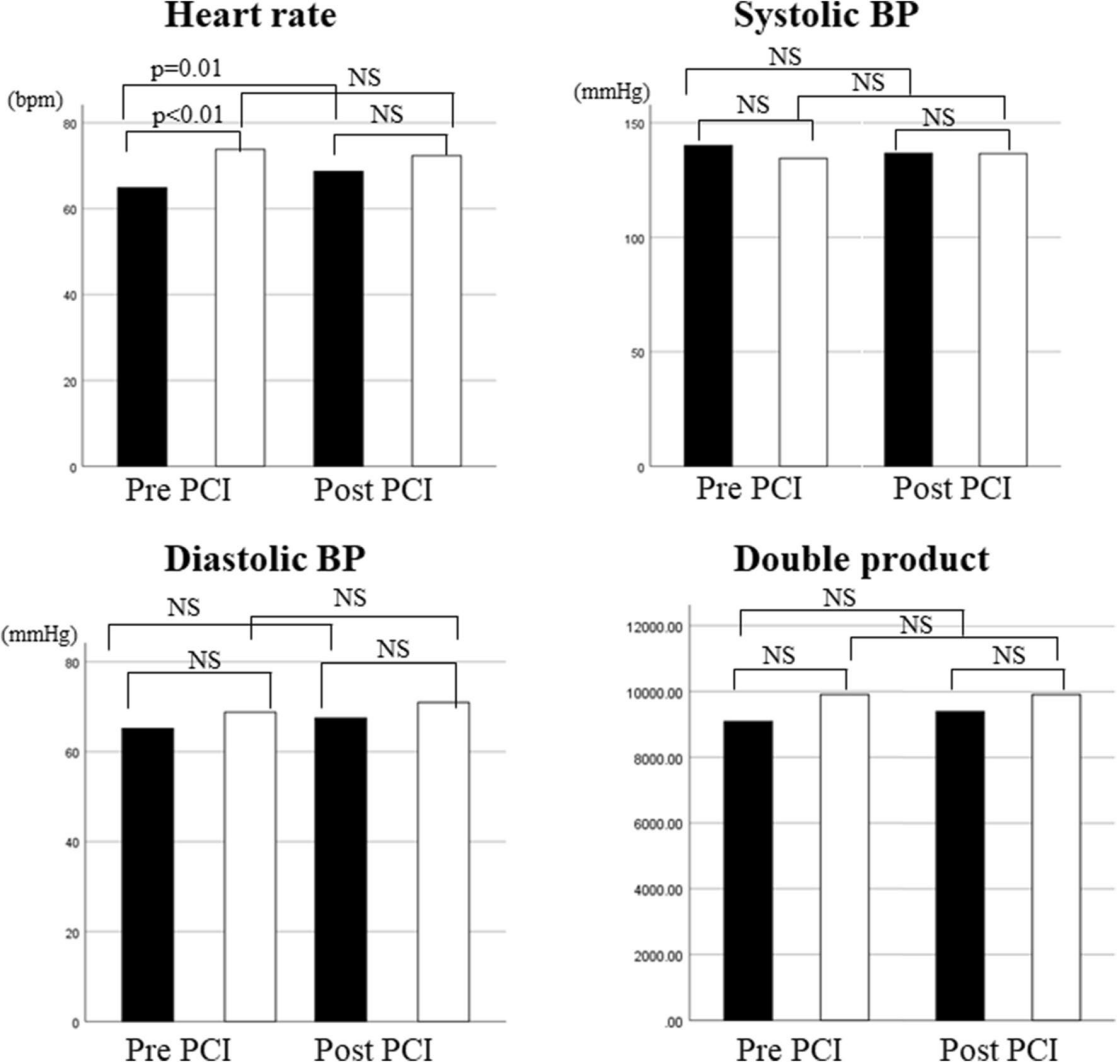
Changes in the heart rate, systolic blood pressure (BP), diastolic BP, and double product after percutaneous coronary intervention in the poor concordance group (black boxes) and in the good concordance group (open boxes). For statistical evaluation, see Table 5. *NS* not significant, *BP* blood pressure

The good concordance and poor concordance groups consisted of 42 and 28 patients, respectively. Regarding the baseline clinical characteristics, the average age was significantly higher in the poor concordance group than in the good concordance group (75.9 ± 9.1 vs. 70.7 ± 10.9 years, *p* = 0.049) (Table 1). In terms of lesion characteristics, the proportion of the left anterior descending artery tended to be larger in the poor concordance group (*p* = 0.052). There was no significant difference between the two groups in either angiographic lesion classification or physiological lesion classification (Table 2). QCA parameters were similar between the two groups (Table 3). On the contrary, IVUS image analysis showed that the pre-intervention proximal ref LA tended to be smaller and pre-intervention MLA was significantly smaller in the poor concordance group than in the good concordance group (7.08 ± 2.19 vs. 8.25 ± 2.53 mm^2^, *p* = 0.053; 1.73 ± 0.46 vs. 2.12 ± 0.83 mm^2^, *p* = 0.038, respectively), while the MSA and stent diameter were significantly smaller and stent length tended to be longer in the poor concordance group than in the good concordance group (4.61 ± 1.62 vs. 5.72 ± 1.66 mm^2^, *p* = 0.005; 2.82 ± 0.36 vs. 3.02 ± 0.42 mm, *p* = 0.037; 29.14 ± 11.93 vs. 23.36 ± 11.92 mm, *p* = 0.051, respectively) (Table 4). Regarding the hemodynamics shown in Table 5, the poor concordance group had a significantly lower HR before PCI and longer physiological LL (PLL) than those in the good concordance group (64.9 ± 10.3 vs. 73.8 ± 10.2 beats/min, *p* = 0.001, 27.69 ± 9.86 mm vs. 20.45 ± 11.81 mm, *p* = 0.009), while the systolic and diastolic BPs and double product were similar in the two groups. After PCI, the HR in the poor concordance group was similar to that in the good concordance group, but significantly higher than that before PCI in the poor concordance group (64.9 ± 10.3 vs. 68.7 ± 11.6 beats/min, *p* = 0.01), while the HR did not statistically change in the good concordance group (73.8 ± 10.2 vs. 72.4 ± 24.2 beats/min, *p* = 0.179). There were no statistically significant changes after PCI in other hemodynamic parameters: systolic and diastolic BP, and double product (DP) in each group (Table 5 and Fig. 5). Moreover, a two-way ANOVA also showed that PCI affected the change in HR between the two groups (*p* = 0.003), but not in other parameters. Considering the independence of the variables that were found to be statistically significant predictors of poor concordance based on univariate analysis, multivariate logistic regression analysis demonstrated that pre-intervention HR was the only independent predictor of poor concordance (odds ratio, 0.936; 95% confidence interval 0.883–0.992; *p* = 0.027) (Table 6). Moreover, the difference between the post-iFR and iFRpred, which was a continuous dependent variable, was analyzed by SEM as part of multivariate analysis, after which the Pearson’s correlation coefficient revealed the following predictors: the MSA, PLL, and RD. A path diagram of the multivariate analysis by SEM showed that only the MSA tended to negatively correlate with the difference between the post-iFR and iFRpred, showing that the standardized regression coefficient of the MSA variable was − 0.25 (*p* = 0.054). However, all 3 covariances (MSA, PLL, and RD) as predictors were significant to each other, whereby the goodness-of-fit index for the structural model validity decreased (goodness-of-fit index = 0.756; root-mean-square error of approximation = 0.273).

**Table 1:** Baseline clinical characteristic

	Good (*n* = 42)	Poor (*n* = 28)	*p*
Age, years	70.7 ± 10.9	75.9 ± 9.1	0.049
Male, *n* (%)	33 (78.5)	20 (71.4)	0.690
Hypertension	35 (83.3)	27 (96.4)	0.129
Dyslipidemia	33 (78.6)	20 (71.4)	0.690
Family history	7 (16.7)	2 (7.1)	0.423
Diabetes	15 (35.7)	14 (50.0)	0.347
Hyperuricemia	8 (19.0)	5 (17.9)	1.00
CKD	17 (40.5)	13 (46.4)	0.650
Smoking	24 (57.1)	13 (46.4)	0.525
Prior MI	16 (38.1)	9 (32.1)	0.799
MI area	7 (2.4)	1 (3.8)	0.192
Multivessel disease	19 (45.2)	13 (46.4)	0.478
LV hypertrophy	11 (26.2)	11 (39.3)	0.372
%LVEF (%)	65.8 ± 11.3	62.4 ± 14.1	0.269

*Good* good concordance group, *Poor* poor concordance group, *CKD* chronic kidney disease, *MI* myocardial infarction, *LVEF* left-ventricular ejection fraction

**Table 2:** Lesion characteristics

	Good (*n *= 42)	Poor (*n* = 28)	*p*
Target vessels, *n* (%)			
LMT	2 (4.8)	1 (3.6)	0.052
LAD	27 (64.3)	25 (89.3)	
Dg	0 (0.0)	1 (3.6)	
RCA	9 (21.4)	1 (3.6)	
LCx	4 (9.5)	0 (0.0)	
De novo	37(88.1)	25 (89.3)	1.00
Angiographic lesion classification			
Focal	20 (47.6)	15 (46.4)	0.660
Tandem	20 (47.6)	11 (39.3)	
Diffuse	2 (4.8)	2 (7.1)	
Physiological lesion classification			
Focal	23 (54.8)	12 (42.9)	0.364
Tandem	11 (26.2)	9 (32.1)	
Focal/gradual	4 (9.5)	1 (3.8)	
Gradual	4 (9.5)	6 (21.4)	

*Good* good concordance group, *Poor* poor concordance group, *LMT* left main trunk, *LAD* left anterior descending coronary artery, *Dg* diagonal branch, *RCA* right coronary artery, *LCx* left circumflex coronary artery

**Table 3:** Quantitative coronary analysis

	Good (*n *= 42)	Poor (*n* = 28)	*p*
Preprocedure			
Ref. diameter (mm)	2.79 ± 0.58	2.53 ± 0.63	0.757
MLD (mm)	1.12 ± 0.44	0.90 ± 0.39	0.350
%DS (%)	61.52 ± 15.20	64.19 ± 13.67	0.331
Postprocedure			
MLD (mm)	2.69 ± 0.52	2.64 ± 0.65	0.512
%DS (%)	8.75 ± 12.76	7.65 ± 16.28	0.118

*Ref. diameter* reference diameter, *MLD* minimum lumen diameter; *%DS* %diameter stenosis

**Table 4:** Intracoronary imaging parameters

	Good (*n *= 42)	Poor (*n* = 28)	*p*
Preprocedure			
Prox.ref.area (mm^2^)	8.25 ± 2.53	7.08 ± 2.19	0.053
MLA (mm^2^)	2.12 ± 0.83	1.73 ± 0.46	0.038
MSA (mm^2^)	5.72 ± 1.66	4.61 ± 1.62	0.005
Stent diameter (mm)	3.02 ± 0.42	2.82 ± 0.36	0.037
Stent length (mm)	23.36 ± 11.92	29.14 ± 11.93	0.051

*Good* good concordance group, *Poor* poor concordance group, *Prox.ref.area* proximal reference area, *MLA* minimum lumen area, *MSA* minimum stent area

**Table 5:** Comparison of hemodynamic parameters between good and poor concordance groups

	Good (*n *= 42)	Poor (*n* = 28)	*p*
Preprocedure			
HR (beats/min)	73.8 ± 10.2	64.9 ± 10.3	0.001
Sys BP (mmHg)	134.5 ± 24.7	140.1 ± 20.7	0.288
Dias BP (mmHg)	68.8 ± 14.9	65.1 ± 11.9	0.282
Double product	9903.6 ± 2207.6	9082.4 ± 1892.5	0.105
FFR	0.68 ± 0.09	0.67 ± 0.09	0.950
iFR	0.75 ± 0.18	0.71 ± 0.16	0.366
PLL (mm)	20.45 ± 11.81	27.69 ± 9.86	0.009
ΔiFR	0.17 ± 0.17	0.19 ± 0.15	0.480
iFRpred	0.92 ± 0.06	0.94 ± 0.03	0.112
Postprocedure			
HR (beats/min)	72.4 ± 24.5	68.7 ± 11.6^§^	0.190
Sys BP	136.5 ± 11.0	136.6 ± 21.5	0.983
Dias BP	71.0 ± 16.8	67.5 ± 11.8	0.350
Double product	9909.2 ± 2478.4	9395.3 ± 2199.3	0.366
Post FFR	0.85 ± 0.07 ^§^	0.83 ± 0.06^§^	0.128
Post-iFR	0.92 ± 0.06 ^§^	0.87 ± 0.06 ^§^	< 0.001

*Good* good concordance group, *poor* poor concordance group, *HR* heart rate, *sys BP* systolic blood pressure, *DBP* diastolic blood pressure, *FFR* fractional flow reserve, *iFR* instantaneous free-wave ratio, *PLL* physiological lesion length, *ΔiFR* delta iFR, *iFRpred* predicted iFR, *post* postinterventional

^§^*p* < 0.05 compared with preprocedural parameters

**Table 6:** Multivariate logistic regression analysis for predictors of poor agreement between the actual iFR and predicted iFR after coronary intervention Exp(*B*), exponentiation of the *B* coefficient

	Exp(*B*)	95% CI	*p*
Age	1.047	0.986–1.111	0.137
LAD	2.243	0.464–10.838	0.315
MSA	0.788	0.521–1.192	0.260
Pre-intervention HR	0.936	0.883–0.992	0.027
PLL	1.029	0.978–1.082	0.277

*% CI* 95% confidence interval, *LAD* left anterior descending coronary artery, *MSA* minimum stent area, *HR* heart rate, *PLL* physiological lesion length, *iFR* instantaneous wave-free ratio

### Subanalysis of tandem lesions

Given the peculiarity of the resting flow, a subanalysis was performed for 30 physiological tandem lesions in the present study. The mean physiological interstenosis distance was 23.17 ± 15.23 mm and the mean difference between the iFRpred and post-iFR was − 0.025 ± 0.038. Pearson’s correlation coefficient indicated no significant correlation between these two parameters (*r* = − 0.139, *p* = 0.583). Moreover, among the physiological tandem lesions, the good (*n* = 11) and poor (*n* = 9) concordance groups had statistically similar interstenosis lengths (23.80 ± 17.85 vs. 22.39 ± 12.30 mm, *p* = 0.365).

## Discussion

The main findings of the present study are as follows. First, after using SyncVision, the lesion morphological classifications changed to other types of physiological classifications in 26 (37%) of the total lesions. Second, the post-iFR had a significantly positive correlation with the iFRpred. However, in most cases, the post-iFR was lower than the iFRpred with poor agreement than that in previous reports [14, 15], which could be attributed to iFR gaps or new occurrences of the iFR. Finally, HR and MSA were considered possible predictors of the discrepancy between post-iFR and iFRpred.

### Feasibility of PCI using SyncVision

Changes in the angiographic lesion classification occurred in 37% of lesions of which the most common type was angiographic tandem lesions; 18 of 31 (58.1%) tandem lesions changed to other types of physiological lesion classification. In terms of coronary tandem lesions, it is difficult to evaluate the physiological severity of each lesion, because under hyperemic flow, one stenosis changes the coronary flow, influencing the trans-stenosis pressure gradient and vice-versa [5–7]. Therefore, equations calculating the FFR values of each stenosis have been introduced; however, they are too complicated and time-consuming to be used in clinical practice [5]. In contrast, resting flow, which can maintain constant flow as long as the %DS is less than 80% [8, 9] and the perfusion pressure is within the coronary self-regulation range [19], is pertinent to evaluate the severity of each stenosis in tandem lesions for physiology-guided PCI. In fact, reclassification using SyncVision can reduce the number or length of lesions identified for revascularization, as shown in case 1 in Fig. 1, as previously reported [14, 15]. Moreover, 6 of 35 (17.1%) angiographic focal lesions changed to physiological tandem lesions (Fig. 2). This suggests that SyncVision can identify lesions that should be treated by revascularization, but might be overlooked based on angiographic assessment alone.

### Agreement between the iFR predicted using SyncVision and actual post-intervention iFR values

Notably, in the present study, iFR gaps in the treated segments and new occurrences of iFR gradients in the physiologically non-significant segments in the treated vessels were observed despite of anatomically satisfactory success. This might be associated with the fact that the post-iFR was lower than expected and the agreement between the post-iFR and iFRpred was poorer (0.029 ± 0.099) than those previously reported (mean biases of 0.016 and 0.011) [14, 15]. Therefore, we scrutinized the predictors of this discrepancy using multivariate analysis for both categorical and continuous dependent variables. This suggested that the pre-intervention HR was significant and MSA had a tendency toward discrepancy. Based on these findings, there are several possible explanations for this discrepancy. First, the diastolic coronary flow cannot completely recover after the removal of stenosis by coronary intervention with stent implantation. iFR is the calculated flow ratio based on a particular period of diastole. Phasic coronary flow is influenced by the obstruction of coronary flow: distal to a coronary stenosis, the diastolic component of phasic coronary flow decreases and returns to a normal diastolic-dominant pattern after the removal of the stenosis by coronary intervention [20]. However, mechanical dilatation such as PCI might not necessarily eliminate the obstruction of coronary flow or normalize the coronary flow due to the residual plaque burden or properties including calcification [21]. In this context, it is relevant that the SEM analysis revealed a tendency of MSA toward the discrepancy between post-iFR and iFRpred in the present study. However, mechanical stent expansion is limited, especially in small arteries, as the present study showed that optimal stent implantation could not be achieved in 15.7% of the cases. Moreover, little is known about the optimal cut-off of the MSA for physiological optimization of stent implantation. Regarding the stent length, which tended to be longer in the poor concordance group but not significantly in the present study, the length of the stent has previously been reported as one of the risk factors for the presence of the residual iFR gradient [22]. In that study, a few iFR measurement points were manually selected, focusing on the culprit lesion without coregistration with angiograms. In this method, the information of the distribution of the iFR gradient across the entire vessel was opaque and the stent length might have conferred a greater effect on the residual iFR gradient than in the present study. The second reason is the influence of the HR. Statistically speaking, the pre-intervention HR was lower in the poor concordance group than in the good concordance group in this study. This does not mean that pre-intervention HR was a direct predictor of poor concordance. As shown in Table 5 and Fig. 5, the HR in the poor concordance group significantly increased after PCI (64.9 ± 10.3 vs. 68.7 ± 11.6 beats/min, *p* = 0.01), while the HR in the good concordance group did not statistically change (73.8 ± 10.2 vs. 72.4 ± 24.2 beats/min, *p* = 0.179). In the poor concordance group, increases in resting heart rates after PCI resulted in higher cardiac work and consequently, increased coronary blood flow [23, 24]. As a result, the changed resting flow after PCI, which was greater than before the PCI, yielded lower post-iFR values than the predicted post-iFR values. On the other hand, in the good concordance group, the predicted iFR value was likely to be close to the actual post-iFR value, because the pre- and post-PCI HRs were similar, which meant that the resting flow did not change. This explains the discordance between the predicted and actual post-iFR observed in the present study. Therefore, we should consider the HR, especially the change in the HR after PCI when the post-iFR is lower than expected, and whether the patient is in a resting state. The third issue is the change in coronary flow after PCI for severe stenotic lesions. When stenoses are severe (FFR value < 0.6), a significant increase in the resting flow of the wave-free period was observed after PCI [25]. Based on these findings, cases of severe stenosis might no longer involve resting flow after PCI. In the present study, the mean pre-interventional FFR value was 0.67 ± 0.09, which was lower than that previously reported [14, 15]. This means that the coronary blood flow increased after PCI, as shown in a previous study [25], which could create or exaggerate the iFR gradient through the mildly narrowed lesion in the vessel and to which iFR gaps or new pressure gradients could be attributed to. Moreover, in high-grade stenoses than those previously reported, an increment in the coronary flow after PCI may occur. The fourth possible explanation is intramyocardial volume reduction. Basically, phasic coronary flow is not affected by left-ventricular pressure as long as the intramyocardial volume can be maintained [26]. However, when the myocardial volume is reduced because of factors such as dehydration, blood loss, or severe stenosis, which can cause collapse of intramyocardial capillaries, the phasic coronary flow becomes ventricularized, showing a remarkably decreasing diastolic component and low iFR value regardless of epicardial coronary disease, which cannot be corrected by PCI. The fifth issue is the occurrence of distal embolism after PCI. A previous study showed that distal embolism reduces the diastolic component of coronary blood flow [27]. Although the entire study population underwent angiographically successful PCI and new ischemic ECG changes, including new abnormal Q waves, were not observed in the present study, serial cardiac troponin values were not measured and evidence of myocardial injury as periprocedural myocardial infarction was not deniable. Therefore, it is possible that distal embolism occurred after PCI, which caused reduced diastolic flow and lower post-iFRs than expected. Finally, in the poor concordance group, the mean post-FFR value was greater than 0.8, although the mean post-iFR was less than 0.89. This means that the degree of improvement in FFR and iFR after PCI is dissociated (Table 5). Although FFR is specific for epicardial coronary stenosis, it is contrary that iFR is not, providing information on microvascular disease as well as epicardial disease [25]. The dissociation between post-FFR and post-iFR could be related to the discordance between predicted and actual post-iFR.

### Tandem lesions

First, the present study was performed based on the assumption that the iFR, which is the resting index, is suitable for assessing the severity of tandem lesions. The present study demonstrated that neither angiographic nor physiological tandem lesions were associated with poor concordance, as were any other types of lesions (Table 2). Moreover, the interstenosis length did not affect the difference between the iFRpred and post-iFR. Considering these findings, iFR pullback assessment of the severity of coronary disease using SyncVision is relevant regardless of lesion morphology or the distance between stenoses.

### Limitations

The present study had several limitations. The study included a small population and was a single-institution study. Large-scale, multicenter studies are required. Although the cut-off of the difference between the post-iFR and iFRpred to allocate the good and poor concordance groups was defined according to the previous reports [14, 15], this dichotomous endpoint may be of concern for the reliability and validity of this study. Hence, we performed SEM for the difference between the post-iFR and iFRpred, which are continuous values, as a dependent variable. However, there were many confounders for which model fitting was mediocre, and no statistical significance was found. As previously mentioned, the iFR, which is measured during diastole of the cardiac cycle, is affected by many factors including the severity of stenosis, HR, and PCI procedure. Therefore, the question remains as to whether resting flow after PCI is no longer resting flow. It is a pressing issue that there is no methodology to confirm resting flow, while the steady state of the maximum hyperemic coronary flow can be confirmed based on a subsequent saline bolus intracoronary injection or change in another hyperemic agent. In addition, we used IVUS and OCT/OFDI; however, these imaging modalities could not be advanced distally in the investigated vessels, and therefore, the intravascular images could not correspond to physiological maps. Therefore, quantitative and qualitative evaluations of coronary plaques were lacking in the present study. Finally, we mentioned the possibility of the relationship between the dissociation of improvement of physiological indexes after PCI and discordance between the predicted and post-iFR. However, we did not further investigate the relationship between iFR and FFR in the present study. Additionally, there were no data supporting this issue in the present study.

## Conclusions

SyncVision, which uses resting flow and coregistration with angiography, revealed that angiographic lesion classification changed to other types of physiological lesion classification in 26 (37%) lesions. It was observed that the post-iFR was significantly related to the iFRpred. However, it was lower than the iFRpred with poor agreement compared to that of the previous reports due to the residual iFR or new occurrences of the iFR, because it is susceptible to changes in the phasic coronary flow. In clinical practice, this imaging-guided therapy device, which is not affected by the coronary flow of adjacent stenotic lesions, can not only avoid unnecessary PCI but also identify lesions requiring treatment; however, it is a caveat for the interpretation of post-iFR.
